# Now and Then I Question Me

**Published:** 1891-03

**Authors:** 


### NOW AND THEN I QUESTION ME.

From the German of Friedrick Bodenstedt.

Now and then I question me,
What hath drawn me so to thee :
Is't your voice so plaintiff, sweet,
Is't your mind with grace replete ?

What doth bind me prisoner here,
When your song is swelling clear ?
Is't your music that enchants,
Or your eye's enticing glance ?

It is not your form nor beauty,
Nor your glance of love and duty,
Nor your voice, though sweetest ever,—
It is all of them together.

